# ASPH Is a Metastatic Factor and Therapeutic Target in Chondrosarcoma

**DOI:** 10.3390/cancers17060951

**Published:** 2025-03-12

**Authors:** Xiaojuan Sun, Jesse Hart, Ross Taliano, Janine Molino, Joseph H. Schwab, Sjoerd Nota, Katsuya Nagaoka, Songhua Zhang, Mark Olsen, Rolf Carlson, Jack Wands, Richard M. Terek

**Affiliations:** 1 Department of Orthopaedics, Warren Alpert Medical School of Brown University, Providence, RI 02903, USAjanine_molino@brown.edu (J.M.); 2 Departments of Pathology, Warren Alpert Medical School of Brown University, Providence, RI 02903, USA; jhart5@lifespan.org (J.H.); rtaliano@lifespan.org (R.T.); 3 Lifespan Biostatistics, Epidemiology, Research Design and Informatics, Rhode Island Hospital, Providence, RI 02903, USA; 4 Department of Orthopaedic Surgery, Harvard Medical School, Massachusetts General Hospital, Boston, MA 02115, USA; joseph.schwab@cshs.org (J.H.S.);; 5 Liver Research Center, Rhode Island Hospital, Warren Alpert Medical School, Brown University, Providence, RI 02903, USA; knagaoka@kumamoto-u.ac.jp (K.N.); rolf_carlson@brown.edu (R.C.);; 6 Pharmacometrics Center of Excellence, Midwestern University, Downer’s Grove, IL 60515, USA; molsen@midwestern.edu; 7 Department of Pharmaceutical Sciences, Midwestern University, Glendale, AZ 85308, USA; 8 Providence Veterans Administration Medical Center, Providence, RI 02908, USA; 9 Legorreta Cancer Center, Brown University, Providence, RI 02903, USA

**Keywords:** small molecule inhibitor, metastasis, bone cancer, sarcoma, chondrosarcoma, targeted therapy, ASPH knockout, precision medicine

## Abstract

**Background:** Chondrosarcoma (CS) is a highly aggressive primary malignant bone tumor for which there are no effective systemic treatments. We assessed aspartate β-hydroxylase (ASPH) as a potential treatment target. ASPH is a transforming cell surface receptor, but its role in chondrosarcoma has not been evaluated. Our goals were to analyze the expression of ASPH in conventional chondrosarcoma, evaluate its utility as a biomarker, and determine if ASPH inhibition diminishes tumor progression in a preclinical model. **Methods:** An annotated tissue microarray was constructed with conventional chondrosarcoma tissues. ASPH expression was quantified with immunohistochemistry. A small molecule inhibitor (SMI) designed to inhibit ASPH activity was evaluated in two CS cell lines with intact ASPH expression and after knockout. Cell viability, invasion, and matrix metalloproteinase (MMP) expression were measured. A mouse xenograft chondrosarcoma model was used to evaluate the effect of the SMI on tumor growth, MMP activity in tumors, and lung metastatic burden. **Results:** Higher ASPH scores were associated with a greater risk of death and metastasis. The SMI decreased CS cell proliferation, invasion, and secretion of MMPs in vitro, and the effects were lost after ASPH knockout. In vivo, systemic administration of the SMI decreased tumor growth, MMP activity and content in xenograft tumors, and lung metastatic burden. **Conclusions:** These data validate ASPH as a biomarker in CS and as a factor in the metastatic phenotype. Systemic treatment with an SMI directed against ASPH inhibits tumor progression in a preclinical model, suggesting that ASPH-targeted therapy may be a new treatment strategy for chondrosarcoma expressing ASPH.

## Simple Summary

Chondrosarcoma (CS) is a highly aggressive primary malignant bone tumor without effective systemic treatments. Our findings identified aspartate β-hydroxylase (ASPH) as a biomarker in CS, as a factor in the metastatic phenotype, and as a potential treatment target for chondrosarcoma. Higher ASPH expression scores and tumor grade were equivalently associated with a greater risk of death and metastasis. A small molecule inhibitor (SMI) of ASPH decreased CS cell proliferation, invasion, and secretion of MMPs in vitro, and the effects were lost after ASPH knockout. In vivo, the SMI decreased tumor growth, MMP activity and content in xenograft tumors, and lung metastatic burden. Our results suggest that ASPH-targeted therapy may be a new treatment strategy for chondrosarcoma.

## 1. Introduction

Conventional chondrosarcoma (CS) is the second most common bone sarcoma. The five-year survival rate for higher grade tumors is 10–25% [1,2]. Surgical resection is the mainstay of treatment and is largely effective for local control. However, unlike in other bone sarcomas, conventional cytotoxic chemotherapy is not effective, and CS patients frequently succumb to pulmonary metastases [3]. Despite the universally poor outcomes, there has been no progress in treatment over the last several decades [4,5]. Thus, there is a need for new treatment strategies for this disease [6].

An alternative or complementary approach to cytotoxic chemotherapy is to inhibit progression with targeted therapeutics. We propose that one such target is aspartate β-hydroxylase (ASPH). ASPH is an oncofetal protein expressed in embryonic development and then in some tumors re-expressed during oncogenesis. Overexpression of ASPH stimulates cell proliferation, migration, invasion, and metastasis. Therefore, ASPH is proposed to generate and maintain malignant phenotypes in cancer. Human ASPH was originally cloned by the immunoscreening of cDNA libraries prepared from human osteosarcoma and hepatocellular carcinoma (HCC) cell lines [7,8]. Subsequently, many splice isoforms of ASPH have been identified. It is well established that ASPH isoform 1 is expressed developmentally, minimally expressed in normal tissues, and then re-expressed at high levels in carcinomas [7,8,9,10]. Other isoforms are expressed in normal tissues, but they lack the C-terminus that contains the transforming hydroxylase activity [11]. Only isoform 1 has hydroxylase activity. We have previously shown that ASPH is expressed in some carcinomas, and ASPH blockade in preclinical models of these malignancies inhibits tumor progression [12]. However, carcinomas are derived from the ectoderm. The cancer biology underlying carcinoma and sarcoma, the latter being derived from mesoderm, is quite different. Therefore, we are interested in studying ASPH in a bone sarcoma model to determine if there is an analogous paradigm. While the regulation of ASPH expression in tumors has yet to be fully determined, its restricted expression at high levels in tumors provides a potential treatment target. Fortuitously, small molecule inhibitors (SMIs) of ASPH have been synthesized that block the enzymatic activity and downstream signaling of ASPH, allowing for the study of ASPH inhibition in preclinical models, although these SMIs are not yet available in the clinic.

Our goal is to analyze the expression of ASPH in conventional chondrosarcoma, evaluate its utility as a biomarker, and determine if ASPH inhibition diminishes tumor progression in a preclinical model.

## 2. Materials and Methods

Cell culture and chemical reagents: Normal human chondrocytes isolated from adult articular cartilage (CH) were cultured in DMEM/F12; short-term culture of cells isolated from a human grade II CS (CSII), and of a patient-derived xenograft (PDX) from the same tumor, PDX as organoids, and human chondrosarcoma cell line CS-1, which was derived from a grade III CS (a gift from Dr. Francis Hornicek, Harvard Medical School, Boston, MA, USA) were cultured in RPMI 1640; human chondrosarcoma cell line JJ (a gift from Dr. Joel Block, Rush Medical School, Chicago, IL, USA) was cultured in complete medium. In addition, 10% FBS and penicillin/streptomycin (100 units/100 μg/mL) were added to the culture media, and cells were cultured in a humidified incubator (NuAire Inc., Plymouth, MN, USA) under 5% CO_2_ as previously described [13]. Cell lines were periodically authenticated using short tandem repeat (STR) profiling (cat# 135-XV, ATCC, Manassas, VA, USA) and tested for mycoplasma contamination (Universal Mycoplasma Detection Kit, cat# rep-mys-20, InvivoGen, San Diego, CA, USA).

Small molecule inhibitors (SMIs) that decrease the β-hydroxylase activity of ASPH were designed and synthesized (MO) based on the crystal structure of the ASPH catalytic site and screened as previously described [14]. The compounds MO-I-1151 and MO-I-1182 were used in this study based on their higher binding affinities to the ASPH catalytic site and inhibitory effects in other models.

Antibodies: Immunohistochemistry (IHC) was performed with the FB-50 mouse monoclonal antibody, which binds to the N-terminus of ASPH and was produced as previously described [8]. For Western blotting, primary antibodies were rabbit polyclonal anti-ASPH antibodies prepared against the full-length human recombinant protein (21st Century Biochemicals, Inc., Marlboro, MA, USA); and anti-GAPDH (MA5-15738, ThermoFisher Scientific). Primary antibodies were detected using a fluorescently labeled secondary IRDye 680RD Donkey anti-rabbit antibody (cat#926-68073) or IRDye 800CW Goat anti-mouse antibody (925-32210) (all secondary antibodies from LI-COR Biosciences).

### 2.1. Chondrosarcoma Tissue Microarray (TMA) and Immunohistochemistry

Patient demographics and details of the TMA were previously described [15]. ASPH expression was only analyzed in conventional CS (50) and not the dedifferentiated CS, a rare subtype. Information about these 50 cases is in Table 1. Briefly, the TMA included duplicate cores from representative regions of tumor blocks from 52 patients with conventional CS, plus 3 lung metastases from 3 of the patients. Tissues from two of the cases detached from the slide and could not be evaluated. In addition, there were 8 benign cartilage tumors (enchondroma) and normal human control tissues (spleen, articular cartilage, liver, lymph node). IHC staining was performed on unstained tissue sections using FB-50 (1:4000), as previously reported [12]. IHC was scored by three investigators in a blinded fashion (JH, XS, RMT): 0 (no staining), 1+ (weak), 2+ (moderate), or 3+ (strong) based on intensity and percentage of cell staining. Scores were averaged for the two sections per case.

### 2.2. Cell Viability Assay:

Cells were seeded on a 96-well plate at 5 × 10^3^ and 1 × 10^3^ cells/well for 3-day and 4-day assays, respectively. Cells were incubated with SMIs at concentrations and for times as listed. The number of viable cells was determined by measuring ATP levels (CellTiter-Glo, cat# G7572, Promega, Madison, WI, USA) according to the manufacturer’s instructions. Briefly, 25 μL of CellTiter-Glo reagents in 100 μL of culture media was added to the cultures, and after 10 min, bioluminescence was measured with a plate reader (Molecular Devices LLC., San Jose, CA, USA).

### 2.3. Invasion Assay

The invasion assay was performed as previously described [13]. Briefly, 5 × 10^4^ cells suspended in serum-free medium were seeded in the upper chambers of 24-well transwell plates (BD Biosciences, Bedford, MA, USA), which were separated from the lower chambers with a Matrigel-coated membrane, 8 μM pore size (Millipore Sigma, Burlington, MA, USA). Medium with 10% FBS was added to the lower chamber. Then, 9 μM MO-I-1182 or control (DMSO) was added to both chambers and cultured for 72 h. The membrane was stained with 0.25% crystal violet solution for microscopy of the cells on the lower side of the membrane, and cells were quantitated by measuring OD of lysates at 570 nm.

### 2.4. ASPH-Knockout

CRISPR/Cas9 system was used to knock out the expression of the ASPH gene. The single-guide RNA (sgRNA) targeting ASPH sequence was 5′-AACCGAGCATAGTTACCACG-3′ and was cloned into pRSGCCR-U6-sg-CMV-Cas9-2A-TagRFP, (Cellecta, Mountain View, CA, USA). Non-targeting (NT) sgRNA in a Cas9 CRISPR lentiviral vector was used as a negative control (cat# SGCCTL-NT-PX, Cellecta, Mountain View, CA, USA). Lentivirus packaging of CRko-sg_hASPH_1-RFP (ASPH-KO) and NT-Control was completed by the Lentivirus Construct Core (RIH, Providence, RI, USA) and stored at −80 °C.

Cells were infected with lentiviral constructs (ASPH-KO) and NT-Control in the presence of polybrene (8 μg/mL) for 3 days. Successfully infected cells were isolated by sorting RFP-positive cells on a flow cytometer (FACS, Becton Dickinson, Franklin Lakes, NJ, USA). Independent single-cell clones lacking the ASPH protein were expanded, confirmed by Western blot, and then used for biological assays.

### 2.5. ELISA Assay

Conditioned media (CM) from cultured cells and lysates from homogenized xenograft and PDX organoid tumors were analyzed for matrix metalloproteinase (MMP) content with ELISA as previously described [13]. Briefly, cells were treated with SMI or control (DMSO) for 48 h; then, the medium was changed to 1% FBS O/N, and the CM were collected. Pro-MMP1 and MMP9 concentrations were measured in duplicate (R&D system, Minneapolis, MN, USA) and normalized to the total protein concentration (Quick Start Brandford, Bio-Rad, Hercules, CA, USA).

### 2.6. Western Blot Analysis

Cell lysates from normal human chondrocytes (CH), chondrosarcoma cell lines (CS-1 and JJ), and short-term culture of cells from CSII and from PDX were analyzed with Western blotting as previously described [13]. Protein concentrations were determined in lysates using a Quick Start Bradford protein assay (Bio-Rad). Briefly, 20 µg of protein was loaded and separated on a mini-SDS-PAGE gel (Bio-Rad). After incubating with primary and secondary antibodies, membranes were scanned on a Licor Odyssey Scanner (LI-COR Biosciences, Lincoln, NE, USA), and quantification was performed with the ImageJ software version 1.48v (National Institutes of Health, USA).

### 2.7. Mouse Models

A CS-1 xenograft model was used to analyze the effect of the SMI in vivo as previously described [13]. Briefly, 1 × 10^6^ CS-1 cells in 100 μL culture medium mixed with 300 μL Matrigel™ (BD Biosciences, San Jose, CA, USA) were injected subcutaneously into the backs of nude mice (nu/nu 6–8-week-old, female, Charles River Laboratory, Wilmington, MA, USA). MO-I-1182 (10 mg/kg) prepared in DMSO or control (DMSO alone) was administered to mice by intraperitoneal injection on 5 consecutive days per week over a four-week period starting one week after implantation of chondrosarcoma cells.

A PDX model was created by implanting 5 mm^3^ pieces of primary human chondrosarcoma tissue subcutaneously into the backs of nu/nu mice. Tumors were harvested, minced, and cultured as organoids and treated with MO-I-1182 or control.

#### 2.7.1. Bioimaging

In vivo bioimaging was performed with fluorescence molecular tomography (FMT, PerkinElmer, Waltham, MA, USA) before harvesting the tumors. Twenty-four hours before imaging, mice were injected via tail vein with 2 nmol MMPSense 680 (PerkinElmer, Waltham, MA, USA). MMPSense content in xenograft tumors was determined by region of interest analysis as previously described [13].

#### 2.7.2. Primary Tumor Analysis

Tumor weight was determined at the time of euthanasia after excision and analyzed with IHC and ELISA.

#### 2.7.3. Metastasis Analysis

Lungs were harvested and lung metastatic burden was quantified as previously described [13]. Briefly, lungs were evaluated with microscopy after fixation in 10% formalin and embedding in paraffin with the ventral side down to ensure consistent sectioning. Coronal sections were obtained via microtomy at 350 µm intervals yielding 18 +/−3.9 SD sections per lung. Hematoxylin and eosin-stained slides were scanned (Philips Ultra-Fast Scanner, Philips, Amsterdam, The Netherlands), and metastatic burden was quantified as the proportion of sections with metastases.

### 2.8. Statistical Analysis

#### 2.8.1. TMA

Descriptive statistics were obtained for all the study variables (Table 1). Mean and standard deviation were reported for the normally distributed continuous variables, median and interquartile range were reported for the nonnormally distributed continuous variables, and frequency and percentage were reported for the categorical variables. Receiver operating curves (ROC) were examined for each of the study outcomes (death, metastasis, and local recurrence). Youden’s J statistic was used to find an optimal cut-point for ASPH score for discriminating between study outcomes. Spearman correlation coefficients, contingency tables, Fisher exact tests, and logistic regression models were estimated to understand the relationship between the study outcomes and (1) ASPH scores (as a categorical measure based on the ROC calculated threshold) and (2) grade. The c-statistics from the logistic regression models were used to assess the discriminatory power of ASPH and grade for each of the study outcomes. Kaplan–Meier curves were estimated for each of the study outcomes to understand the survival functions by ASPH score and grade. The survival functions for each stratum were compared using the likelihood ratio test. Cox regression analysis was used to compare the time to (1) death, (2) metastasis, and (3) local recurrence by ASPH score and grade. Hazard ratios, along with their 95% confidence intervals, are reported. Statistical analysis was performed with SAS version 9.4 (SAS Institute Inc., Cary, NC, USA). For all analyses, a *p*-value < 0.05 was used to determine statistical significance.

#### 2.8.2. Experimental

In vitro experiments were repeated with at least 3 biologic and 3–4 technical replicates. Data are presented as means ± SD. Experiments with three or more groups were compared with one-way ANOVA, followed by Student’s *t*-test for individual comparisons with Bonferroni correction. Experiments with two groups were analyzed with Student’s *t*-test.

Generalized linear models were used to compare mice who received MO-I-1182 or the control. The negative binomial distribution was used for analysis of MMPSense probe content and tumor weight. The negative binomial distribution is positively skewed with distinct parameters for central tendency and variance, as well as having no negative values (14). The binomial distribution was used for analysis of metastatic burden. Statistical analysis was performed with Prism version 5.04 (GraphPad, San Diego, CA, USA) and SAS version 9.4 (SAS Institute Inc., Cary, NC, USA). The null hypothesis of no difference was rejected at a significance level of 5%.

## 3. Results

### 3.1. ASPH Expression in Chondrosarcoma Predicts Survival

**Figure 1:**
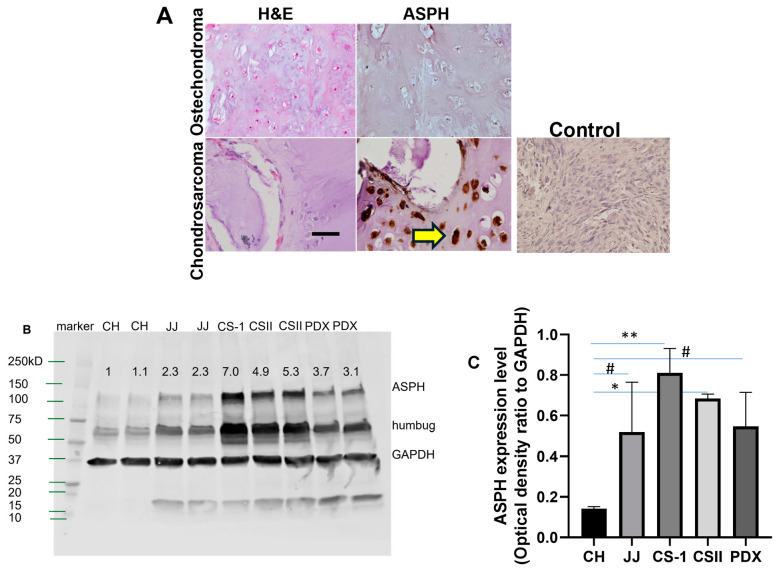
ASPH is expressed in chondrosarcoma. (**A**) Representative H&E staining and IHC with FB-50 Ab shows no ASPH expression in a benign osteochondroma, but +3 staining in a grade II chondrosarcoma (yellow arrow). Bar = 50 µm, 100×. Control is IHC with an antibody against HBV which shows no staining. (**B**) Western blot with ASPH antibody of cell lysates from chondrocytes (CH), chondrosarcoma cell lines (JJ, CS-1), grade II CS cells (CSII), and PDX cells (PDX) (optical density ratio to GAPDH relative to CH lane 1). (**C**) ASPH expression (optical density ratio to GAPDH), analyzed with ImageJ version 148.v. * *p* < 0.001, ** *p* < 0.01, # *p* < 0.05, n = 3.

As a first step in evaluating ASPH expression in CS, we performed IHC of an annotated TMA and additional cartilage tumors. Representative immunohistochemical stains of cartilage tumors are shown in Figure 1A. This grade II CS had +3 staining, whereas the benign osteochondroma had no staining. Lung metastases also had high staining scores (+2.8, n = 3), and enchondroma had low staining (+0.9, n = 6). IHC of PDX also stained positive for ASPH (Figure S2). ASPH expression was also analyzed in normal primary chondrocytes, chondrosarcoma cells and cell lines, and PDX with Western blot. Only the chondrosarcoma cells, cell lines, and PDX express ASPH at a high level (Figure 1B,C). Other isoforms of ASPH such as humbug are also expressed and recognized by the polyclonal antibody used for Western blotting. We then analyzed the ASPH staining score of the primary chondrosarcomas on the TMA as a discriminator of study outcomes.

ROC Analysis: the ROC analysis yielded an optimal cut-point value of ASPH staining of 1.5 for death and metastasis (Youden’s J statistic for death = 0.42, metastasis = 0.38).

Relationship between ASPH scores and Grade: ASPH scores, as both a continuous and binary measure, were positively correlated with grade, Spearman correlation coefficients *r* = 0.67 and *r* = 0.62, respectively, *p* < 0.0001.

Association of ASPH score and grade on Death, Metastasis, and Local Recurrence: Contingency tables and Fisher exact tests were used to assess the effect of ASPH and grade on the study outcomes. Those who were deceased were more likely to have an ASPH score of greater than or equal to 1.5 or grade 2 or 3 (*p* < 0.02 and *p* < 0.009). Patients who experienced metastasis were more likely to have grade 2 or 3 and an ASPH score of greater than or equal to 1.5 (*p* = 0.053 and *p* = 0.04). The distribution of grade and ASPH score did not significantly differ by local recurrence status (Table 2, Table 3 and Table 4).

Discriminatory power of ASPH and grade: Logistic regression models were used to assess the predictive power of ASPH score (<1.5 vs. ≥1.5) and grade for the study outcomes using a c-statistic of 0.7 or above as the criterion for acceptable discriminatory power. ASPH and grade had comparable discriminatory power for death (c-statistic = 0.71 and 0.72, respectively). Grade had acceptable discrimination power for metastasis (c = 0.732, although ASPH had c = 0.69). Neither variable had acceptable discriminatory power for local recurrence.

**Figure 2:**
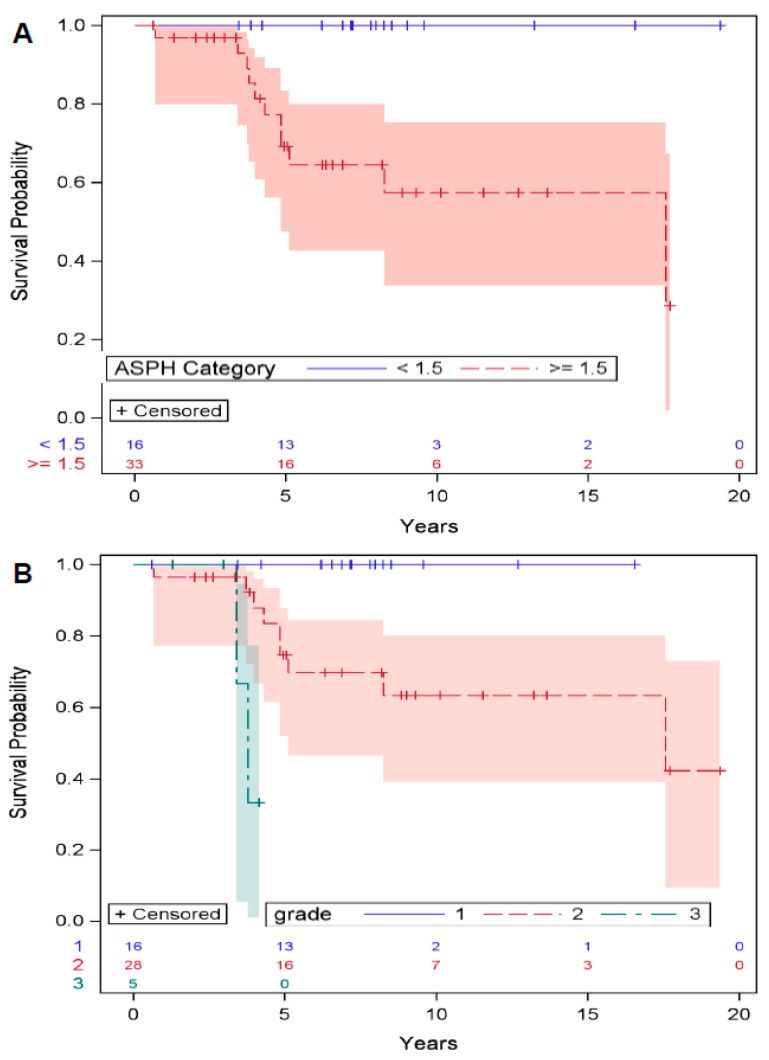
High ASPH expression predicts poor survival. Kaplan–Meier survival curves of patients with chondrosarcoma by intensity of immunostaining with FB-50 mAb: low < 1.5, high ≥ 1.5–3 and grade (I, II, III) (n = 49). (**A**,**B**) The survival function for death significantly differed by ASPH (*p* = 0.0008) and grade (*p* = 0.004), with ASPH ≥ 1.5 and grade ≥ 2 having lower rates of survival. Numbers above X axis indicate number of patients in each group at each time point.

Survival functions: Kaplan–Meier curves were used to understand the survival functions for death, metastasis, and local recurrence by ASPH score (<1.5 vs. ≥1.5) and grade. The survival function for death significantly differed by ASPH (*p* = 0.0008) (Figure 2A) and grade (*p* = 0.004) (Figure 2B), with ASPH ≥ 1.5 and grade ≥ 2 having lower rates of survival. Similarly, the survival function for metastasis significantly differed by ASPH (*p* = 0.004) (Figure S1A) and grade (*p* = 0.007) (Figure S1B). The survival functions for local recurrence did not significantly differ across ASPH and grade strata.

Cox regression: Cox regression analysis was used to assess the risk of death, metastasis, and local recurrence by ASPH and grade. Higher ASPH scores and higher grades were associated with a greater risk of death (HR > 999, 10.4) and metastasis (HR > 999, 4.9). There was no association between local recurrence and ASPH or grade. In summary, ASPH expression and grade are equivalent predictors of metastasis and death, but not local recurrence. Thus, ASPH is a biomarker for high-risk tumors and a potential treatment target.

### 3.2. ASPH Inhibition Decreases CS Proliferation and Invasion In Vitro

**Figure 3:**
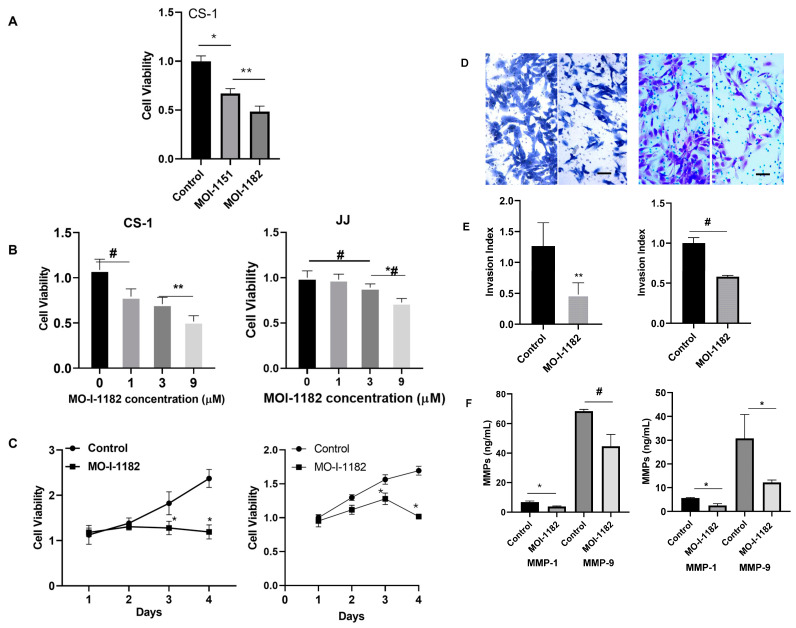
MO-I-1182 inhibits cell viability, invasion, and MMP secretion. Cell viability, invasion, and MMP secretion were determined in chondrosarcoma cells after exposure to SMIs as indicated. (**A**) Viability of CS-1 cells after treatment with MO-I-1182 or MO-I-1151 (10 µM, 3 days) and normalized to control (* *p* < 0.001. ** *p* < 0.05; n = 3). (**B**) Viability of CS-1 and JJ cells after exposure to MO-I-1182 at doses listed for 3 days (CS-1: # *p* < 0.01, ** *p* < 0.05, n = 4; JJ: * *p* < 0.001, # *p* < 0.01, *^,^# *p* < 0.0001, n = 4). (**C**) Viability of CS-1 and JJ cells after treatment with MO-I-1182 (9 µM) for 1-4 days, normalized to control on day 1 (CS-1: # *p* < 0.01, ** *p* < 0.05, n = 4; JJ: * *p* < 0.001, n = 4). (**D**,**E**) Representative images of CS-1 and JJ cells after treatment with MO-I-1182 (9 µM) transgressing a membrane (control left, MO-I-1182 right, 10×, bar = 100 µm) and (**E**) invasion index (** *p* < 0.05, n = 3; # *p* < 0.01, n = 3). (**F**). MMP1 and 9 in conditioned media of CS-1 and JJ cells were measured with ELISA. CS-1: * *p* < 0.001. # *p* = 0.01; n = 3; JJ: * *p* < 0.001, n = 3; ns = not significant.

**Figure 4:**
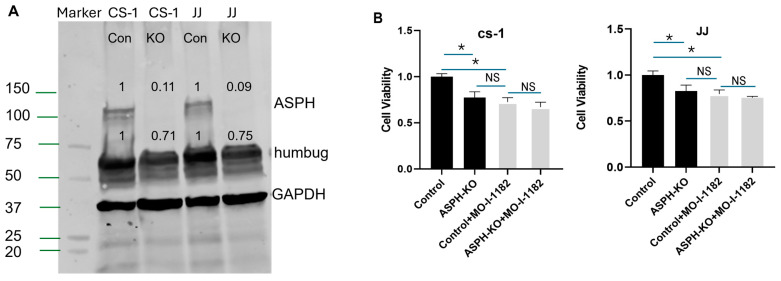
No effect of MO-I-1182 after ASPH KO: (**A**) Western blot with ASPH antibody after CRISPR-Cas9-mediated KO of ASPH in CS-1 and JJ cells. (**B**) Cell viability of CS-1 and JJ cells after ASPH KO and treatment with MO-I-1182 (* *p* < 0.001, n = 4), NS = not significant. Control was NT sgRNA.

We evaluated the effect of ASPH inhibition with the SMIs MO-I-1151 and −1182 on CS cells. Cell viability was decreased by the SMIs, and MO-I-1182 was more effective than -MO-I-1151, 52% vs. 33%, respectively (Figure 3A); hence, −1182 was used in subsequent experiments. MO-I-1182 shows a dose response effect between 0 and 9 µM (Figure 3B). MO-I-1182 flattened the growth curve, inhibiting proliferation in CS-1 by 30% and 50%, and in JJ by 18% and 40%, after three and four days of treatment (9 µM, Figure 3C). In the transwell invasion assay, MO-I-1182 inhibited the invasion index by 62% in CS-1 and 42% in JJ (Figure 3D,E). MMP expression is a known factor in invasion and the metastatic cascade. MMPs 1 and 9 were decreased in conditioned media and organoid PDX tumors after treatment with MO-I-1182 (Figure 3F and Figure S2). CRISPR/Cas9-mediated knockout (KO) of ASPH in both cell lines was confirmed by Western blot (Figure 4A). KO and MO-I-1182 had the same effect on viability, and there was no additional effect of the SMI after KO (Figure 4B), suggesting that ASPH expression positively affects cell viability and that the SMI is specific for ASPH. The KO was specific to ASPH, as humbug was still expressed (Figure 4A).

### 3.3. SMI Inhibits Tumor Growth and Metastasis in Xenograft Chondrosarcoma Model

**Figure 5:**
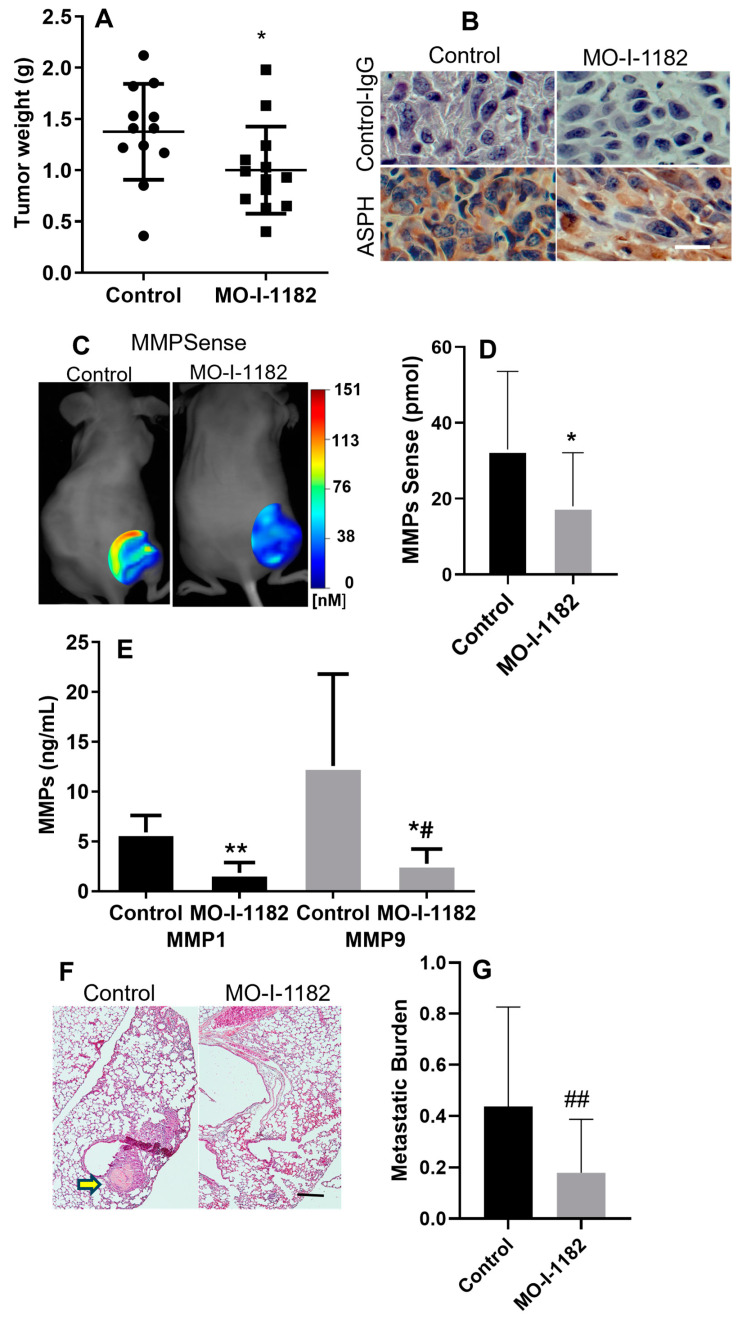
MO-I-1182 inhibits chondrosarcoma progression in xenograft model. Mice with CS-1 xenograft tumors were treated with MO-I-1182 (10 mg/kg i.p., 5 times/week for 4 weeks, n = 13) or DMSO (control, n = 12). (**A**) MO-I-1182 reduced xenograft tumor weight by 27% (* *p* < 0.05). (**B**) IHC of xenograft tumors with control (upper) or FB-50 (lower) antibody (100×, bar = 50 µm). (**C**) FMT bioimaging was performed with MMPSense at week 4. (**D**) MMPSense in xenograft tumors was reduced by MO-I-1182 (* *p* < 0.05, n = 12, 13). (**E**) Total MMP1 and 9 in the tumor lysates measured by ELISA was reduced in the treatment group ** *p* < 0.001. *^,^# *p* = 0.01; n = 8. (**F**) Representative H&E staining of lungs shows metastasis in control (yellow arrow, 4×, bar = 200 µm). (**G**) Metastatic burden (proportion of slides with metastases) was reduced in the treatment group, (## *p* = 0.03, n = 12, 13).

A mouse xenograft chondrosarcoma model was used to assess whether MO-I-1182 could inhibit tumor growth and metastasis in vivo. Tumor weight was significantly lower in the MO-I-1182 group than in the control group (1.00 vs. 1.37, *p* = 0.05, Figure 5A). ASPH expression was confirmed in treated and control xenograft tumors with IHC (Figure 5B). We measured MMP activity in the tumors using in vivo bioimaging and ex vivo analysis of MMP content. MMPSense was significantly lower in the MO-I-1182 group than in the control group (18 vs. 33, *p* = 0.03, Figure 5C,D). MMP1 and 9 contents in xenograft tumors were decreased 68.7% and 78%, respectively, after 4 weeks of treatment (Figure 5E). These results would predict that treatment would decrease lung metastases. Metastatic burden was in fact decreased in the SMI-treated animals, 16.6% (95% CI = 8.1–31.0%) vs. 43.7% (95% CI = 23.9–65.7%), *p* = 0.03 (Figure 5F,G).

### 3.4. SMI Inhibits MMP Expression in PDX Tumors

PDX tumors in culture were used to assess the effect of MO-I-1182 on MMP expression. H&E staining shows that the PDX tumors closely resemble the original tumor (Figure S2A). IHC shows that ASPH was highly expressed in PDX tumors (Figure S2B). MMP1 and 9 were reduced by 44.9% and 72.4%, respectively, after 10 days of treatment with MO-I-1182 (Figure S2C, n = 3, * *p* = 0.001).

Taken together, the results indicate that MO-I-1182 inhibits CS progression through the ASPH pathway.

## 4. Discussion

Our results show that ASPH is expressed in human chondrosarcoma cell lines, primary tumors, PDX, and metastases. ASPH expression in primary tumors is predictive of clinical outcomes, and its inhibition decreases tumor progression in a preclinical model. ASPH expression and grade are predictive of death and metastasis but not local recurrence, which is consistent with the notion that these are distinct biologic processes.

Survival was analyzed with IHC using the FB-50 monoclonal antibody, which recognizes the full-length, enzymatically active ASPH isoform 1 that is associated with malignant transformation, as well as the smaller isoform humbug [16]. Humbug lacks hydroxylase activity but may signal through alternative mechanisms. The IHC results reflect a combination of these ASPH isoforms. While some studies have used ASPH isoform 1-specific antibody for IHC [17], many have used the FB-50 antibody, and staining for ASPH with FB-50 has been shown to negatively correlate with survival in numerous studies of carcinomas; therefore, we used the FB-50 antibody in this study [12,18]. Because the cancer biology underlying carcinoma and sarcoma, the latter being derived from mesoderm, is different, we are surprised to find high expression of ASPH in chondrosarcoma. To our knowledge, this is the first report of ASPH expression in human bone sarcoma specimens, and its restricted expression in tumors provides a potential treatment target.

ASPH is a member of the α-ketoglutarate-dependent dioxygenase family [19]. Its mechanism of action is related to the hydroxylation of aspartyl and asparaginyl residues in epidermal growth factor (EGF)-like repeats of various proteins required for signal transduction. These hydroxylation events in turn activate a number of cancer-related pathways including Notch. One potential mechanism of ASPH blockade in our model is a reduction of Notch signaling. Notch signaling is a well-recognized pathologic feature of cancer. In bone sarcomas, Notch signaling promotes tumor growth and metastasis, in part by transcriptionally upregulating the expression of proteinases such as MMPs [20,21]. Some of the hallmarks of cancer progression such as invasion and metastasis are mediated by expression of MMPs [22]. MMP expression correlates with a poor prognosis in chondrosarcoma and another primary bone tumor, osteosarcoma [23,24]. Therefore, inhibiting MMP expression may have beneficial effects by decreasing tumor spread. Direct inhibition of Notch activation with the use of γ-secretase inhibitors has not been successful clinically due to drug toxicities and lack of targeting [25]. Therefore, SMIs targeted to ASPH are a particularly promising means of indirectly inhibiting Notch signaling.

We used the latest generation SMI developed to inhibit ASPH hydroxylase activity: MO-I-1182 [12]. We show that ASPH blockade with MO-I-1182 inhibits chondrosarcoma cell growth both in vitro and in vivo. It is noteworthy that the SMI inhibited mediators of the metastatic phenotype such as in vitro invasion and MMP activity and content in the cell lines and in xenograft and PDX tumors (Figure 3F, Figure 5C–E and Figure S2C). Most importantly, lung metastatic burden, which is what leads to patients’ demise, is decreased in a xenograft model with MO-I-1182 treatment (Figure 5F,G). Furthermore, the in vitro effects were more dramatic in the higher-ASPH-expressing cell line CS-1 (Figure 1B and Figure 3B,C), and the effect of the drug was lost when ASPH was knocked out in these cells (Figure 4B). Thus, the SMI is specific to ASPH and is not likely to have off-target effects. ASPH KO decreases cell viability, reinforcing its importance in this tumor. Because the FB-50 Ab used for IHC and the polyclonal antibody used for Western blotting also detect humbug, additional strategies would potentially need to be employed to block humbug, as humbug lacks the catalytic site blocked by the SMI and may have independent signaling mechanisms [26].

Traditional treatment of some high-grade sarcomas includes cytotoxic chemotherapy which includes an anthracycline. The consensus is that chemotherapy is not effective for conventional chondrosarcoma [27]. Targeted cancer therapies, which block tumor growth or metastasis by interfering with specific molecular targets, are currently the focus of much anticancer drug development. We have identified the cell surface protein aspartate β-hydroxylase (ASPH) as another molecular target for chondrosarcoma. There are any number of molecular abnormalities and associated targets that have been discovered in CS including isocitrate dehydrogenase (IDH) mutations, upregulation of Indian Hedgehog (IHH) signaling, tyrosine kinase receptor activity, misexpression of microRNAs, overexpression of growth factors and anti-apoptotic proteins, and mutations in tumor suppressor genes [28,29,30]. However, clinical translation has yet to be achieved. Single-agent-targeted therapy in other cancers typically results in modest improvements in survival, but when synergistic combinations of targeted therapies are identified, the results can be more dramatic. Combination therapies can also prevent or delay the onset of resistance to the treatment. We observed modest inhibition of CS progression with MO-I-1182 as a single agent. Additional work needs to be undertaken to optimize dosing and to identify synergistic drug combinations.

## 5. Conclusions

Our results show that ASPH, an oncofetal protein, is expressed in chondrosarcoma and is a predictor of the development of metastasis and death. Inhibition of ASPH with an SMI decreased tumor progression in a preclinical model, suggesting that ASPH-targeted therapy may have potential for chondrosarcoma treatment in ASPH-expressing tumors.

## Figures and Tables

**Table 1:** Patient Demographics.

Patient Demographics	
Male, n (%)	23 (46%)
Age at Presentation, mean (SD)	53.4 (14.7)
Disease Characteristics	
Average ASPH Score, n (%)	
0	0 (0%)
1	16 (32%)
1.5	8 (16%)
2	17 (34%)
2.5	4 (8%)
3	5 (10.0%)
Average ASPH Score, mean (SD)	1.74 (0.65)
ASPH Score ≥ 1.5	34 (68.0%)
Grade, n (%)	
0	0 (0%)
1	16 (33%)
2	29 (57%)
3	5 (10%)
Overall Survival Outcomes	
Died, n (%)	11 (22.5%)
Metastasis, n (%)	8 (16.0%)
Local Recurrence, n (%)	10 (20.0%)
Time to Occurrence	
Months to death, median (IQR)	76.3 (46.7–107.7)
Months to metastasis, median (IQR)	76.3 (46.7–107.7)
Months to local recurrence, median (IQR)	59.1 (35.8–98.9)

**Table 2:** Effect of ASPH score and grade on death.

	Deceased (n = 11)	Alive (n = 48)	*p*-Value
Grade			0.02
0	0 (0%)	0 (0%)	
1	0 (0%)	16 (42.1%)	
2	9 (81.8%)	19 (50.0%)	
3	2 (18.2%)	3 (7.9%)	
ASPH score			0.02
1	0 (0%)	16 (42.1%)	
1.5	4 (36.4%)	4 (10.5%)	
2	5 (45.5%)	11 (29.0%)	
2.5	1 (9.1%)	3 (7.9%)	
3	1 (9.1%)	4 (10.5%)	
ASPH Score			0.009
<1.5	0 (0%)	16 (42.1%)	
≥1.5	11 (100%)	22 (57.9%)	

**Table 3:** Effect of ASPH score and grade on metastasis.

	Metastasis (n = 8)	No Metastasis (n = 42)	*p*-Value
Grade			0.053
0	0 (0%)	0 (0%)	
1	0 (0%)	16 (38.1%)	
2	6 (75.0%)	23 (54.8%)	
3	2 (25.0%)	3 (7.1%)	
ASPH score			0.078
1	0 (0%)	16 (38.1%)	
1.5	3 (37.5%)	5 (11.9%)	
2	4 (50.0%)	13 (31.0%)	
2.5	0 (0%)	4 (9.5%)	
3	1 (12.5%)	4 (9.5%)	
ASPH Score			0.04
<1.5	0 (0%)	16 (38.1%)	
≥1.5	8 (100%)	26 (61.9%)	

**Table 4:** Effect of ASPH score and grade on local recurrence.

	Recurrence (n = 10)	No Recurrence (n = 40)	*p*-Value
Grade			0.77
0	0 (0%)	0 (0%)	
1	2 (20.0%)	14 (35.0%)	
2	7 (70.0%)	2 (55.0%)	
3	1 (10.0%)	4 (10.0%)	
ASPH score			0.25
1	2 (20.0%)	14 (35.0%)	
1.5	4 (40.0%)	4 (10.0%)	
2	3 (30.0%)	14 (35.0%)	
2.5	0 (0%)	4 (10.0%)	
3	1 (10.0%)	4 (10.0%)	
Average ASPH Score			0.47
<1.5	2 (20%)	14 (35.0%)	
≥1.5	8 (80%)	26 (65.0%)	

## Data Availability

The data generated during the current study are available from the corresponding author on reasonable request.

## Supplementary Materials

The following supporting information can be downloaded at https://www.mdpi.com/article/10.3390/cancers17060951/s1, Figure S1. High ASPH expression predicts metastasis. Figure S2. PDX tumors were cultured as organoids.

## References

[1] Damron T.A., Ward W.G., Stewart A. (2007). Osteosarcoma, chondrosarcoma, and Ewing’s sarcoma: National Cancer Data Base Report. Clin. Orthop. Relat. Res..

[2] Lee F.Y., Mankin H.J., Fondren G., Gebhardt M.C., Springfield D.S., Rosenberg A.E., Jennings L.C. (1999). Chondrosarcoma of bone: An assessment of outcome. J. Bone Jt. Surg. Am..

[3] van Maldegem A.M., Bovee J.V., Gelderblom H. (2014). Comprehensive analysis of published studies involving systemic treatment for chondrosarcoma of bone between 2000 and 2013. Clin. Sarcoma Res..

[4] Giuffrida A.Y., Burgueno J.E., Koniaris L.G., Gutierrez J.C., Duncan R., Scully S.P. (2009). Chondrosarcoma in the United States (1973 to 2003): An analysis of 2890 cases from the SEER database. J. Bone Jt. Surg. Am..

[5] Soderstrom M., Ekfors T.O., Bohling T.O., Teppo L.H., Vuorio E.I., Aro H.T. (2003). No improvement in the overall survival of 194 patients with chondrosarcoma in Finland in 1971–1990. Acta Orthop. Scand..

[6] Landuzzi L., Ruzzi F., Lollini P.-L., Scotlandi K. (2025). Chondrosarcoma: New Molecular Insights, Challenges in Near-Patient Preclinical Modeling, and Therapeutic Approaches. Int. J. Mol. Sci..

[7] Korioth F., Gieffers C., Frey J. (1994). Cloning and characterization of the human gene encoding aspartyl beta-hydroxylase. Gene.

[8] Lavaissiere L., Jia S., Nishiyama M., de la Monte S., Stern A.M., Wands J.R., Friedman P.A. (1996). Overexpression of human aspartyl(asparaginyl)beta-hydroxylase in hepatocellular carcinoma and cholangiocarcinoma. J. Clin. Investig..

[9] Jia S., VanDusen W.J., Diehl R.E., Kohl N.E., Dixon R.A., Elliston K.O., Stern A.M., Friedman P.A. (1992). cDNA cloning and expression of bovine aspartyl (asparaginyl) beta-hydroxylase. J. Biol. Chem..

[10] Patel N., Khan A.O., Mansour A., Mohamed J.Y., Al-Assiri A., Haddad R., Jia X., Xiong Y., Megarbane A., Traboulsi E.I. (2014). Mutations in ASPH cause facial dysmorphism, lens dislocation, anterior-segment abnormalities, and spontaneous filtering blebs, or Traboulsi syndrome. Am. J. Hum. Genet..

[11] Zou Q., Hou Y., Wang H., Wang K., Xing X., Xia Y., Wan X., Li J., Jiao B., Liu J. (2018). Hydroxylase Activity of ASPH Promotes Hepatocellular Carcinoma Metastasis Through Epithelial-to-Mesenchymal Transition Pathway. EBioMedicine.

[12] Ogawa K., Lin Q., Li L., Bai X., Chen X., Chen H., Kong R., Wang Y., Zhu H., He F. (2019). Aspartate beta-hydroxylase promotes pancreatic ductal adenocarcinoma metastasis through activation of SRC signaling pathway. J. Hematol. Oncol..

[13] Sun X., Charbonneau C., Wei L., Chen Q., Terek R.M. (2015). miR-181a Targets RGS16 to Promote Chondrosarcoma Growth, Angiogenesis, and Metastasis. Mol. Cancer Res..

[14] Kanwal M., Polakova I., Olsen M., Kasi M.K., Tachezy R., Smahel M. (2024). Heterogeneous Response of Tumor Cell Lines to Inhibition of Aspartate β-hydroxylase. J. Cancer.

[15] Nota S., Al-Sukaini A., Patel S.S., Sabbatino F., Nielsen G.P., Deshpande V., Yearley J.H., Ferrone S., Wang X., Schwab J.H. (2021). High TIL, HLA, and Immune Checkpoint Expression in Conventional High-Grade and Dedifferentiated Chondrosarcoma and Poor Clinical Course of the Disease. Front. Oncol..

[16] Dinchuk J.E., Henderson N.L., Burn T.C., Huber R., Ho S.P., Link J., O’Neil K.T., Focht R.J., Scully M.S., Hollis J.M. (2000). Aspartyl beta -hydroxylase (Asph) and an evolutionarily conserved isoform of Asph missing the catalytic domain share exons with junctin. J. Biol. Chem..

[17] Zhang Y., Gao Y., Li Y., Zhang X., Xie H. (2020). Characterization of the Relationship Between the Expression of Aspartate beta-Hydroxylase and the Pathological Characteristics of Breast Cancer. Med. Sci. Monit..

[18] Gan X., Li S., Wang Y., Du H., Hu Y., Xing X., Cheng X., Yan Y., Li Z. (2023). Aspartate beta-Hydroxylase Serves as a Prognostic Biomarker for Neoadjuvant Chemotherapy in Gastric Cancer. Int. J. Mol. Sci..

[19] Zheng W., Wang X., Hu J., Bai B., Zhu H. (2020). Diverse molecular functions of aspartate beta-hydroxylase in cancer (Review). Oncol. Rep..

[20] Engin F., Bertin T., Ma O., Jiang M.M., Wang L., Sutton R.E., Donehower L.A., Lee B. (2009). Notch signaling contributes to the pathogenesis of human osteosarcomas. Hum. Mol. Genet..

[21] Xu F., Zhang Z.Q., Fang Y.C., Li X.L., Sun Y., Xiong C.Z., Yan L.Q., Wang Q. (2016). Metastasis-associated lung adenocarcinoma transcript 1 promotes the proliferation of chondrosarcoma cell via activating Notch-1 signaling pathway. Oncol. Targets Ther..

[22] Hanahan D., Weinberg R.A. (2011). Hallmarks of cancer: The next generation. Cell.

[23] Berend K.R., Toth A.P., Harrelson J.M., Layfield L.J., Hey L.A., Scully S.P. (1998). Association between ratio of matrix metalloproteinase-1 to tissue inhibitor of metalloproteinase-1 and local recurrence, metastasis, and survival in human chondrosarcoma. J. Bone Jt. Surg. Am..

[24] Zhou J., Liu T., Wang W. (2018). Prognostic significance of matrix metalloproteinase 9 expression in osteosarcoma: A meta-analysis of 16 studies. Medicine.

[25] Zhdanovskaya N., Firrincieli M., Lazzari S., Pace E., Scribani Rossi P., Felli M.P., Talora C., Screpanti I., Palermo R. (2021). Targeting Notch to Maximize Chemotherapeutic Benefits: Rationale, Advanced Strategies, and Future Perspectives. Cancers.

[26] Lee J.H. (2008). Overexpression of humbug promotes malignant progression in human gastric cancer cells. Oncol. Rep..

[27] Jeys L.M., Thorkildsen J., Kurisunkal V., Puri A., Ruggieri P., Houdek M.T., Boyle R.A., Ebeid W., Botello E., Morris G.V. (2024). Controversies in orthopaedic oncology. Bone Joint J..

[28] Walter S.G., Knoll P., Eysel P., Quaas A., Gaisendrees C., Nissler R., Hieggelke L. (2023). Molecular In-Depth Characterization of Chondrosarcoma for Current and Future Targeted Therapies. Cancers.

[29] Chen J.C., Chen M.S., Jiang S.K., Eaw C.Y., Han Y.J., Tang C.H. (2025). Transcriptomic data integration and analysis revealing potential mechanisms of doxorubicin resistance in chondrosarcoma cells. Biochem. Pharmacol..

[30] van Oosterwijk J.G., Herpers B., Meijer D., Briaire-de Bruijn I.H., Cleton-Jansen A.M., Gelderblom H., van de Water B., Bovee J.V. (2012). Restoration of chemosensitivity for doxorubicin and cisplatin in chondrosarcoma in vitro: BCL-2 family members cause chemoresistance. Ann. Oncol..

