# Supplementary material for: ASPH Is a Metastatic Factor and Therapeutic Target in Chondrosarcoma
Source: Cancers (Basel). 2025 Mar 12;17(6):951. doi: 10.3390/cancers17060951 (PMC11939963; doi:10.3390/cancers17060951)
Supplement: Supplementary file 1 [file cancers-17-00951-s001.zip › cancers-3386111-supplementary.pdf]

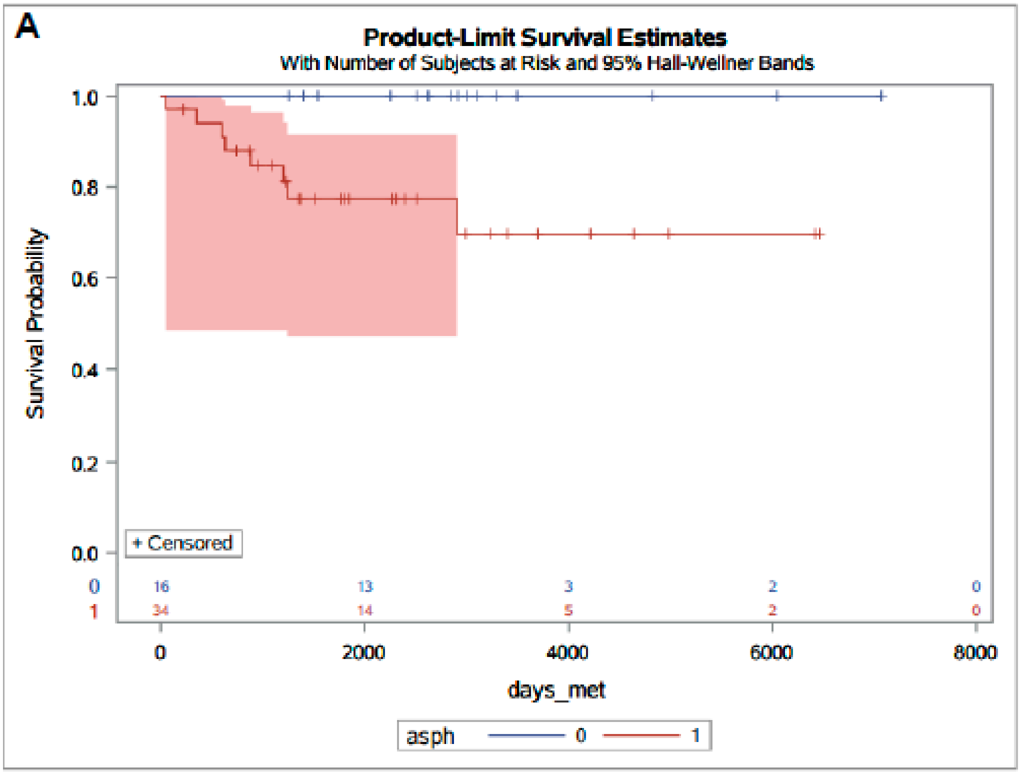

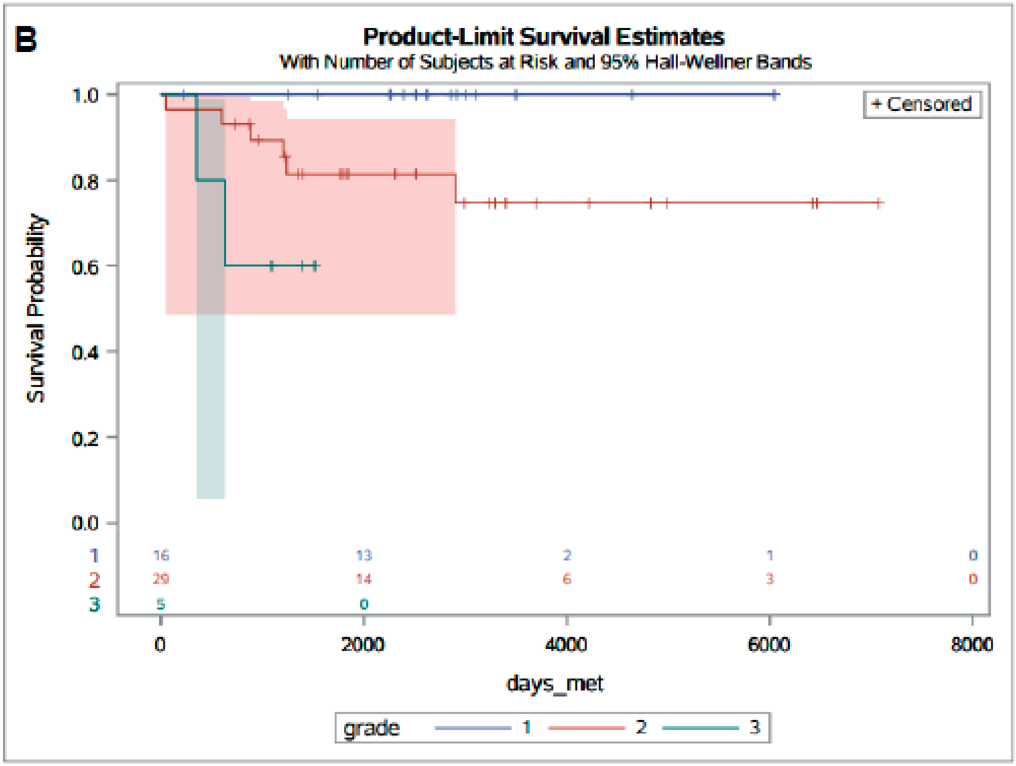

Figure S1. High ASPH expression predicts metastasis. The survival function for metastasis significantly differed by (A) ASPH ($p = 0.004$) and (B) grade ($p = 0.007$), with ASPH ≥ 1.5 and grade ≥ 2 having higher rates of metastasis. Numbers above X axis indicate number of patients in each group at each time point.

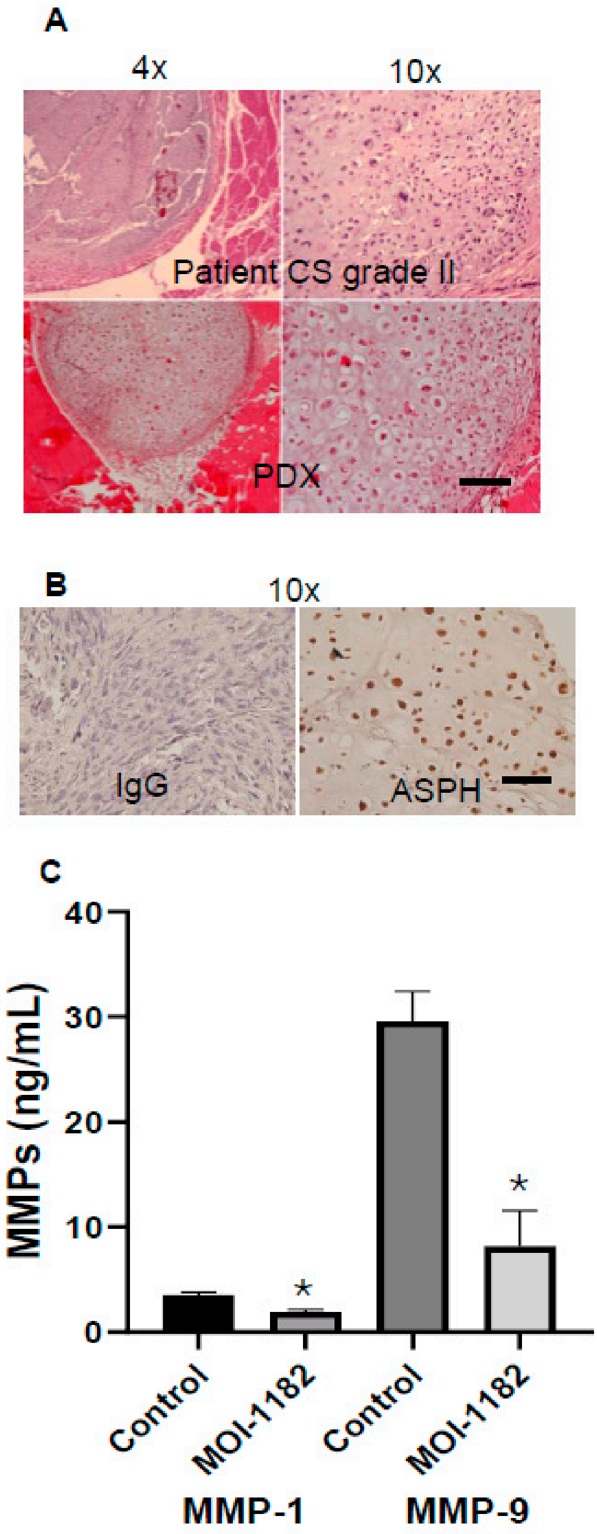

Figure S2. PDX tumors were cultured as organoids. (A) H&E stains of primary and PDX tumors. (B) IHC for ASPH in PDX tumor (FB-50 antibody) bar = 50 µm. (C): MMP1 and 9 in lysates of PDX organoid cultures after treatment with MO-I-1182 (n = 3, *, p = 0.001).
